# Supplementary material for: Shifting patterns of dengue three years after Zika virus emergence in Brazil
Source: Nat Commun. 2024 Jan 20;15:632. doi: 10.1038/s41467-024-44799-x (PMC10799945; doi:10.1038/s41467-024-44799-x)
Supplement: Supplementary file 1 — Supplementary information [file 41467_2024_44799_MOESM1_ESM.pdf]

Supplementary Information for "Shifting patterns of dengue three years after Zika virus emergence in Brazil."

Supplementary Methods

Additional details on population initialisation

Here we present an algorithm to initialise a synthetic population with the desired age distribution and serological profile. The central idea is to divide the procedure in two steps: first, we sample age and residual lifetime for each individual, and then generate an infection history that is compatible with serological data.

The first step consists in sampling age A and residual lifetime R from the joint distribution $p(A, R)$. Let us denote with $f(L)$ and $S(L) = \int_L^{\infty} f(x)dx$, respectively, the probability density and the inverse cumulative distribution of life-expectancy L. In order to sample from $p(A, R)$, we first sample A from $S(A)/\bar{L}$, where $\bar{L}$ is the average life expectancy and then sample L from $f(L)$, conditioned on L being larger than A; finally, $R = L - A$ is the residual lifetime. This procedure guarantees that the population is already at demographic equilibrium at $t = 0$ where the age distribution is the one induced by the life-expectancy distribution $f(L)$.

The second step makes use of a sero-catalytic model to determine the number and timing of prior DENV infections, under the simplifying assumption that serotypes have spread independently in the past; more in detail, we assume that time τ since infection is distributed according to a non-homogeneous Poisson process with intensity $\lambda(x) = \lambda_1$ if $x \leq A_0$ and $\lambda(x) = \lambda_2$ if $x > A_0$. For each individual aged A we then sample four infection times, one for each DENV serotype, and retain only those infection events such that $\tau < A$. Overall, this process implies that the probability of being seronegative at age A is equal to $exp\left(-4\int_0^A \lambda(x)dx\right)$.

Finally, we initialise the status of each individual at $t = 0$. If an individual is seronegative, it is automatically set to susceptible. Otherwise, we need to decide whether the host is incubating, infectious, cross-protected or susceptible after its most recent infection event, which we assume to have happened at time τ in the past due to serotype i. Now, let $t_E \sim Geom(\epsilon_D)$, $t_I \sim Geom(\sigma_D)$ and $t_R \sim Geom(l_D)$: if $t_E > \tau$ we set the individual exposed to i; if not, we check if $t_E + t_I > \tau$, in which case we set the individual infectious with i. If not, we check if $t_E + t_I + t_R > \tau$, in which case we set the individual as cross-protected, after having recovered from i. If this is still not the case, we set the individual as susceptible (on top of being fully immune to i).

Alternatively, it is possible to initialise a population's serological profile in a purely mechanistic way, by letting the population be exposed to DENV serotypes for a long enough period. More in detail, we initialise the age profile as before, but let DENV spread for 100 years before introducing ZIKV and/or collecting any other information. Furthermore, at $t = 0$ we randomly select a proportion $1 - R_{0,D}^{-1}$ of hosts for each DENV serotype and immunise them to that serotype.

Supplementary Figures

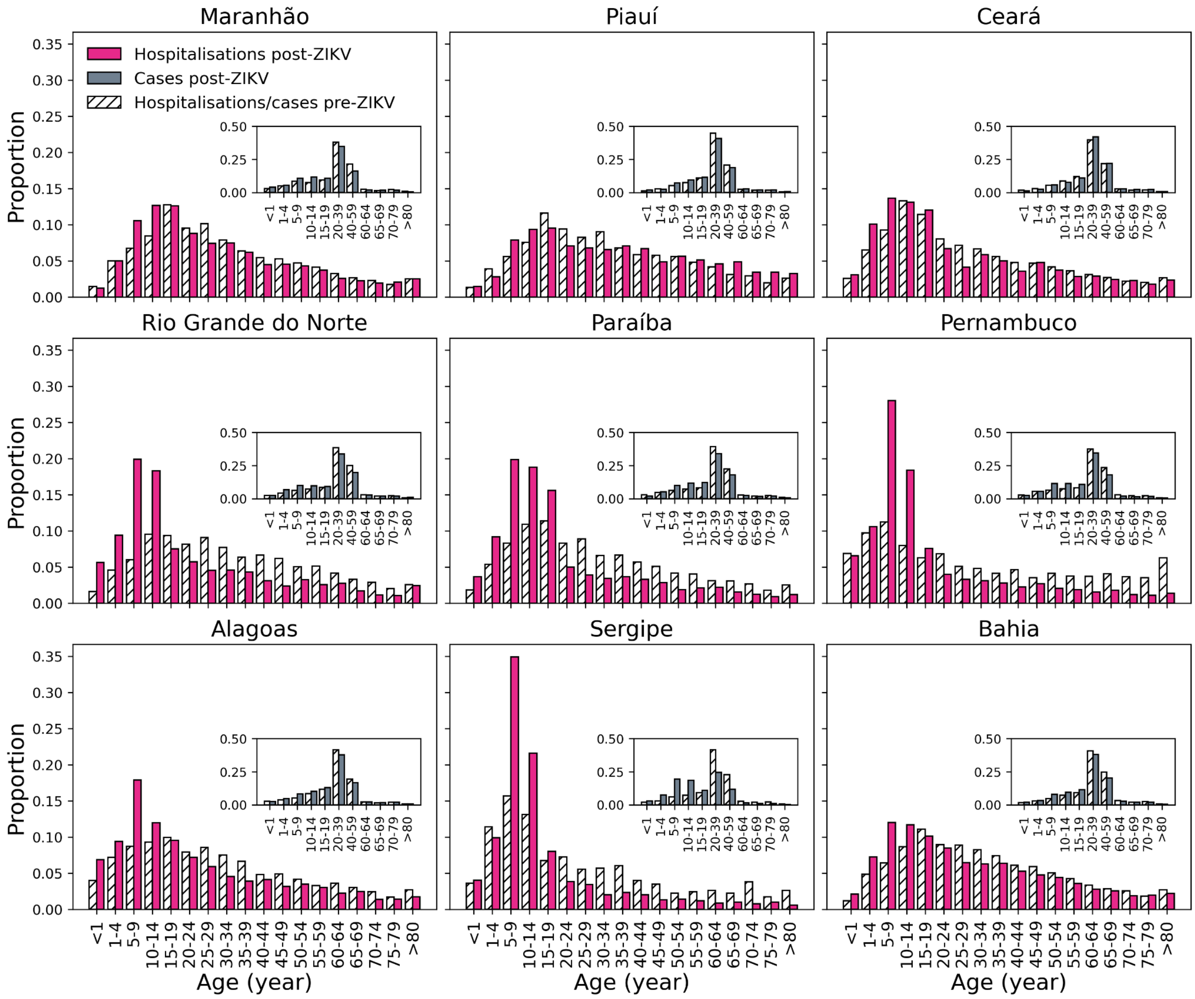

Supplementary Figure 1. **Age distribution of DENV in the North-Eastern region of Brazil.** Each panel shows the age distributions for hospitalisations (fuchsia, main panel), and cases (grey, inset) after ZIKV in a different state in the North-East. The corresponding distribution relative to the pre-ZIKV period is also shown for comparison (hatches). Pre- and post-ZIKV distributions are based on data from the periods 2013-2015 and 2018-2019, respectively.

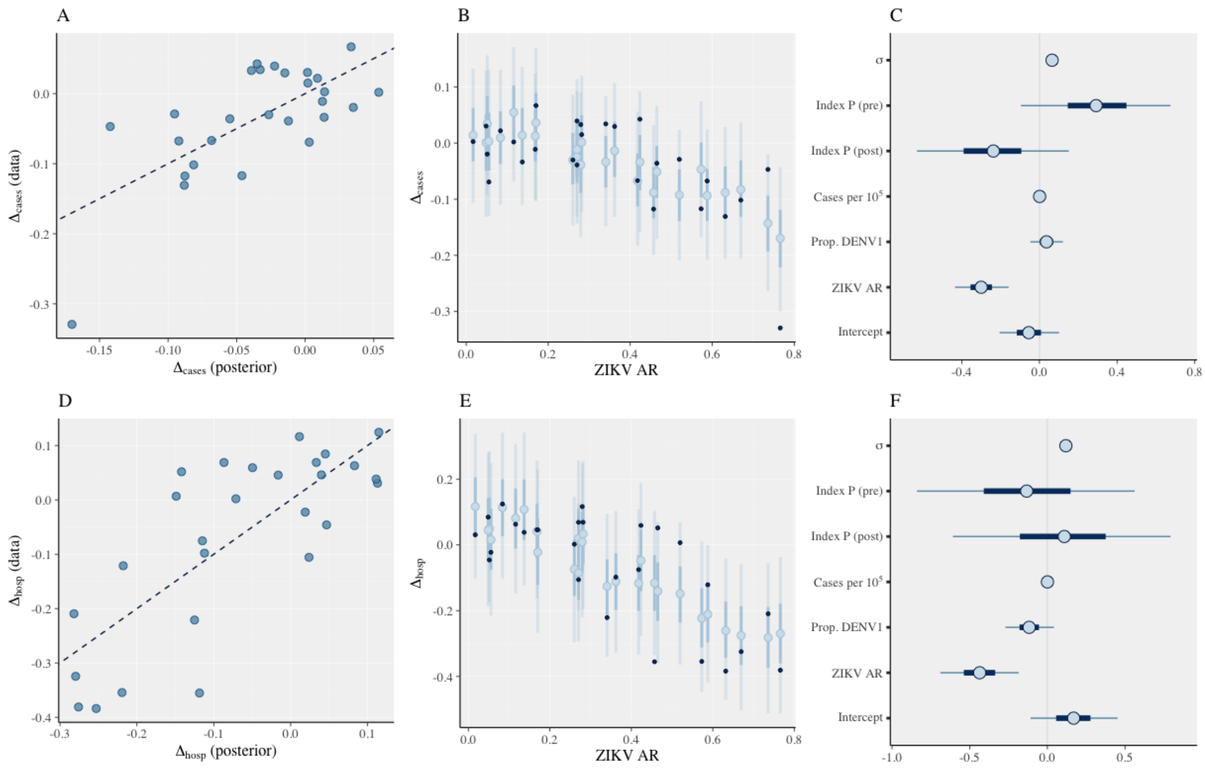

Supplementary Figure 2. **Linear regression analysis.** (A) Observed vs fitted values. The line is a guide for the eye (B) Posterior median (blue circles) and central intervals estimates (50% and 90%) for Δ_{cases} with observed data overlaid (black dots), plotted against ZIKV attack rate. (C) Posterior median and central intervals (50% and 90%) estimates for regression coefficients, intercept term and error standard deviation (σ). (D-F) Same as above, but with Δ_{hosp} as the dependent variable. The regression coefficient relative to estimated ZIKV attack rate is significantly different from 0 for both case and hospitalisation data. Furthermore, ignoring ZIKV attack rate had a significantly negative effect on predictive accuracy, while adding region-specific intercepts did not improve model fit noticeably (not shown). Results are based on 4000 posterior samples collected from 4 independent chains after an initial warmup of 1000 steps. Covariates used for linear regression are described in the Methods section in the main manuscript and links to relevant data are provided in "Data Availability".

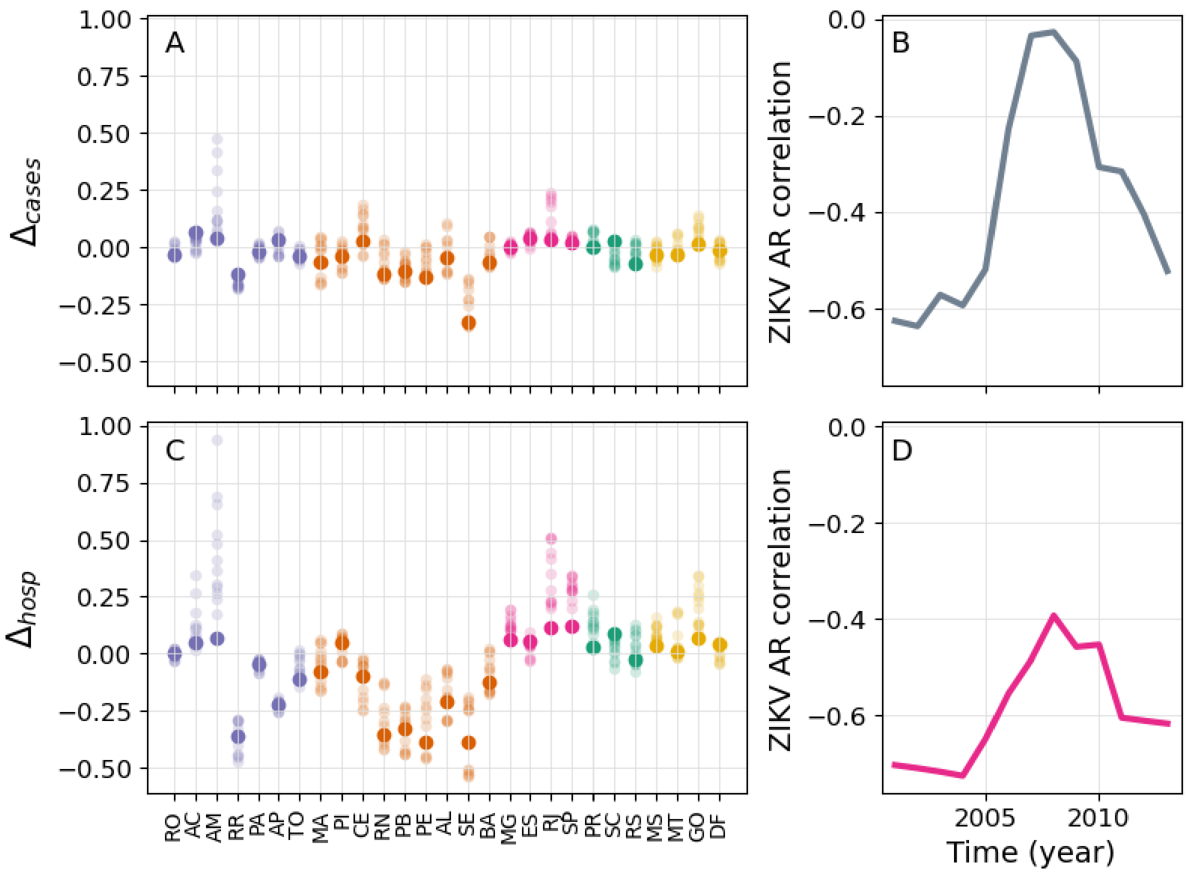

Supplementary Figure 3. **Age variation with respect to different time periods.** (A) We calculate the age-shift $\Delta = (\bar{A}_{post} - \bar{A}_{pre})\,/\bar{A}_{pre}$ for different states and with respect to different reference periods to estimate $\bar{A}_{pre}$; for each state, $\bar{A}_{post}$ is fixed and calculated based on cases reported in the period 2018-2019. Solid circles denote the latest estimate based on the period 2013-2015, while dim circles correspond to earlier periods. (B) Spearman rank correlation coefficient between Δ and ZIKV attack rate using different reference periods to estimate $\bar{A}_{pre}$, indexed by the starting year t; the period itself ends in year $t + 2$. (C,D) Same as panels (A,B), but using hospitalisation data instead of reported cases. Panels suggest that the statistical association between ZIKV attack rate and Δ is robust with respect to the choice of the reference time frame, although the statistical signal is somewhat diminished when the pre-ZIKV period includes years between 2007 and 2010. It should be noted, however, that two serotype changes occurred in 2007 and in 2009, due to DENV2 and DENV1 respectively. These events may partially explain the observed loss in statistical signal.

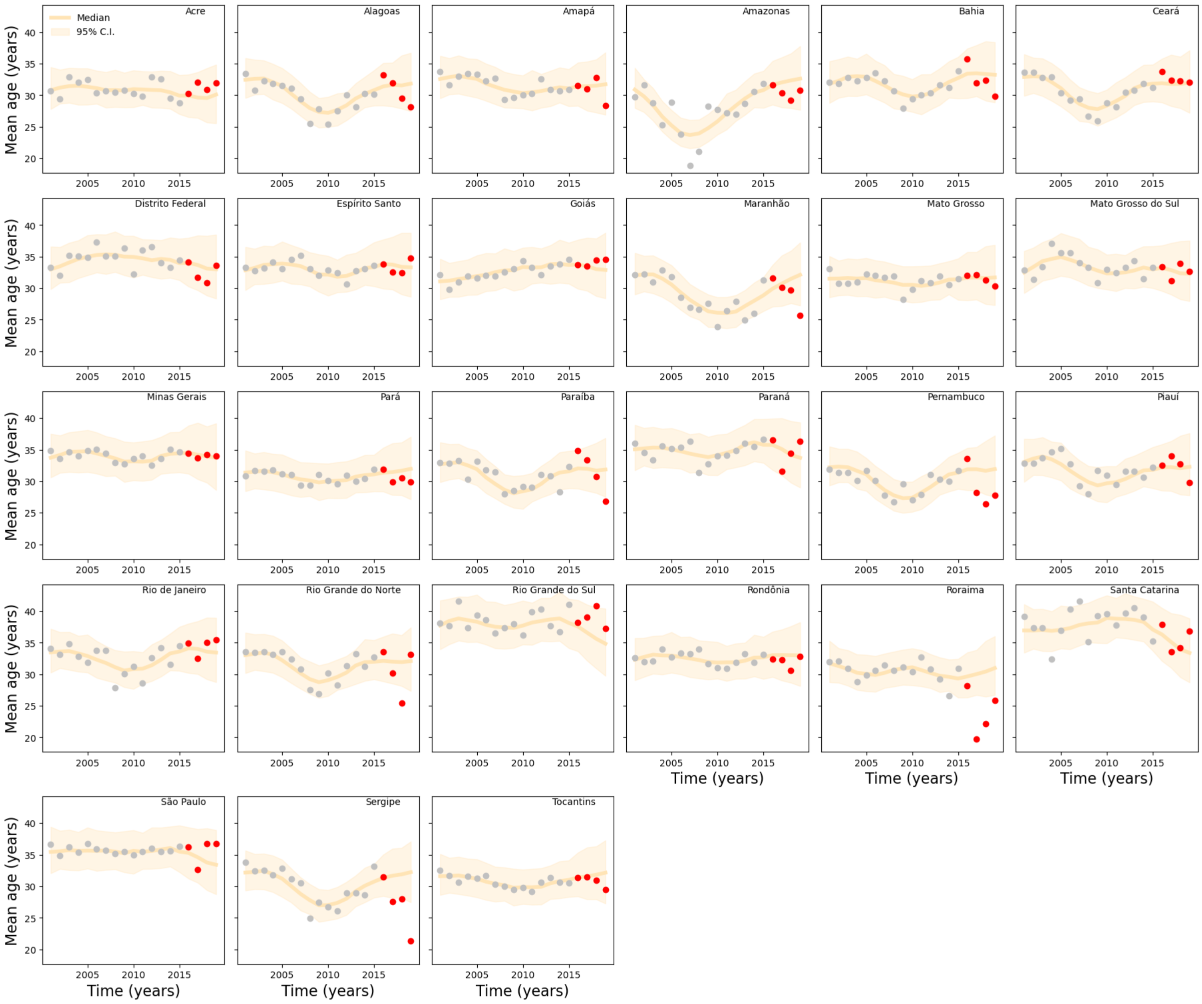

Supplementary Figure 4. **Time series analysis of mean age of DENV cases.** We use gaussian process regression to investigate spatio-temporal trends in the mean age of DENV cases, $\bar{A}_{i,t}$, from $t = 2001$ to $t = 2019$ and across states $i = 1, ..., 27$. Gaussian processes provide a flexible, non-parametric framework to uncover non-linear trends in data[3]. We assume that the logarithm of mean case age $\widehat{A}_{i,t} = log\, \bar{A}_{i,t}$ is distributed according to a gaussian process with zero mean ($\widehat{A}_{i,t}$ is also standardised by subtracting its mean prior to fitting) and covariance function $C \cdot K[\vec{u}_k, \vec{u}_l] + \sigma^2 \delta_{k,l}$, where C is a constant, $\vec{u}_k$ is a vector of covariates consisting of space and time coordinates for observation k and $\sigma^2 \delta_{k,l}$ is a white noise covariance function. Spatial coordinates were calculated for each state using the *representative_point* function in *Geopandas* v0.11.1[4]. We choose K to be a radial basis function kernel with a separate scale parameter for each covariate (3 in total). In order to verify whether pre-ZIKV trends in mean case age of DENV can explain post-ZIKV data, we fit this model to data up to $t = 2015$ (grey scatters) and use the resulting posterior predictive distribution to predict values $\bar{A}_{i,t}$ for $t > 2015$ (red scatters). Lines and shaded areas denote the posterior median and 95% C.I. based on 400 samples from the posterior predictive distribution. Further comments on these results can be found in Supplementary Fig. 6.

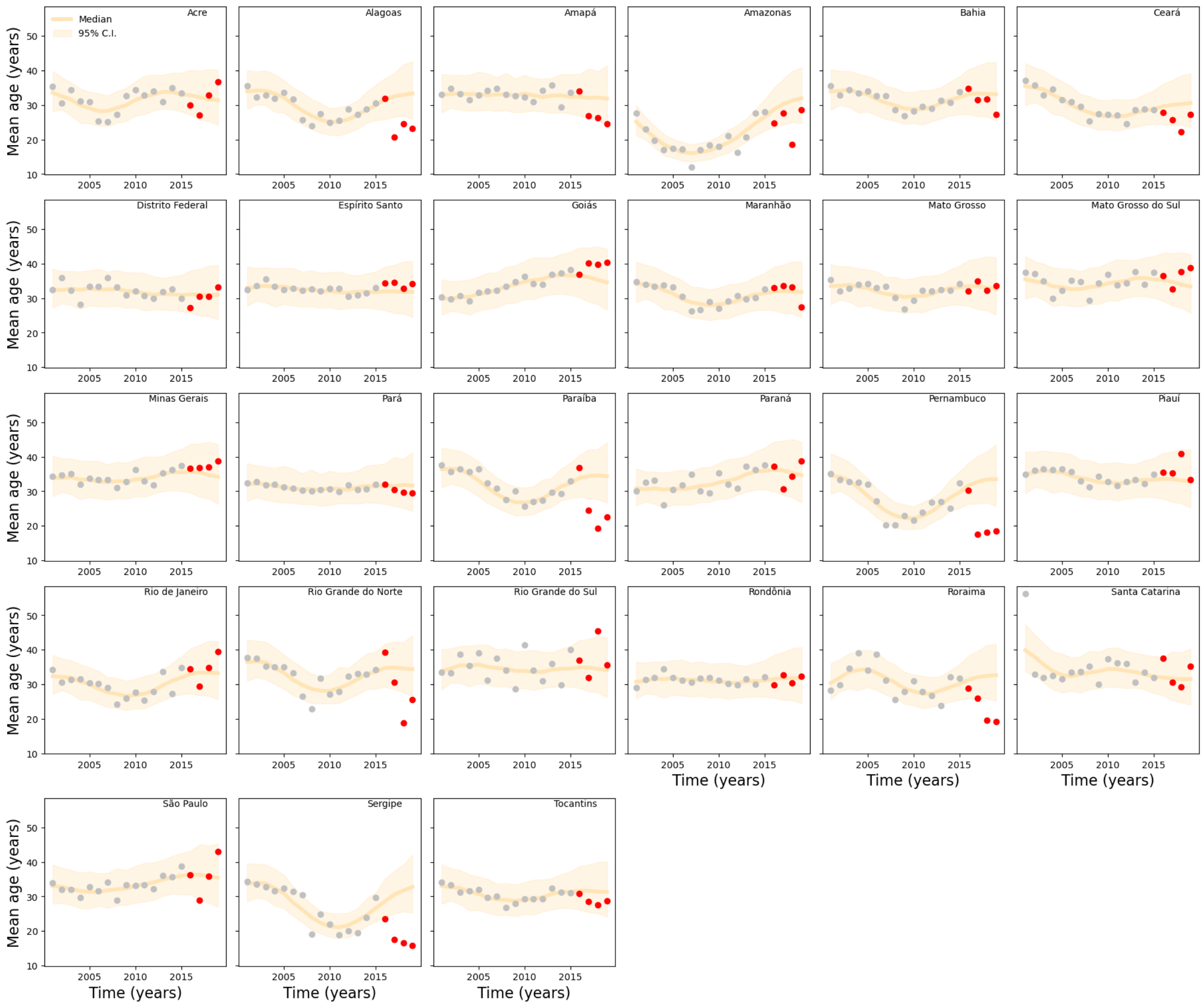

Supplementary Figure 5. **Time series analysis of mean age of DENV hospitalisations.** This figure repeats the analysis of Supplementary Fig. 4 but using hospitalisation data instead of cases. Further comments on these results can be found in Supplementary Fig. 6.

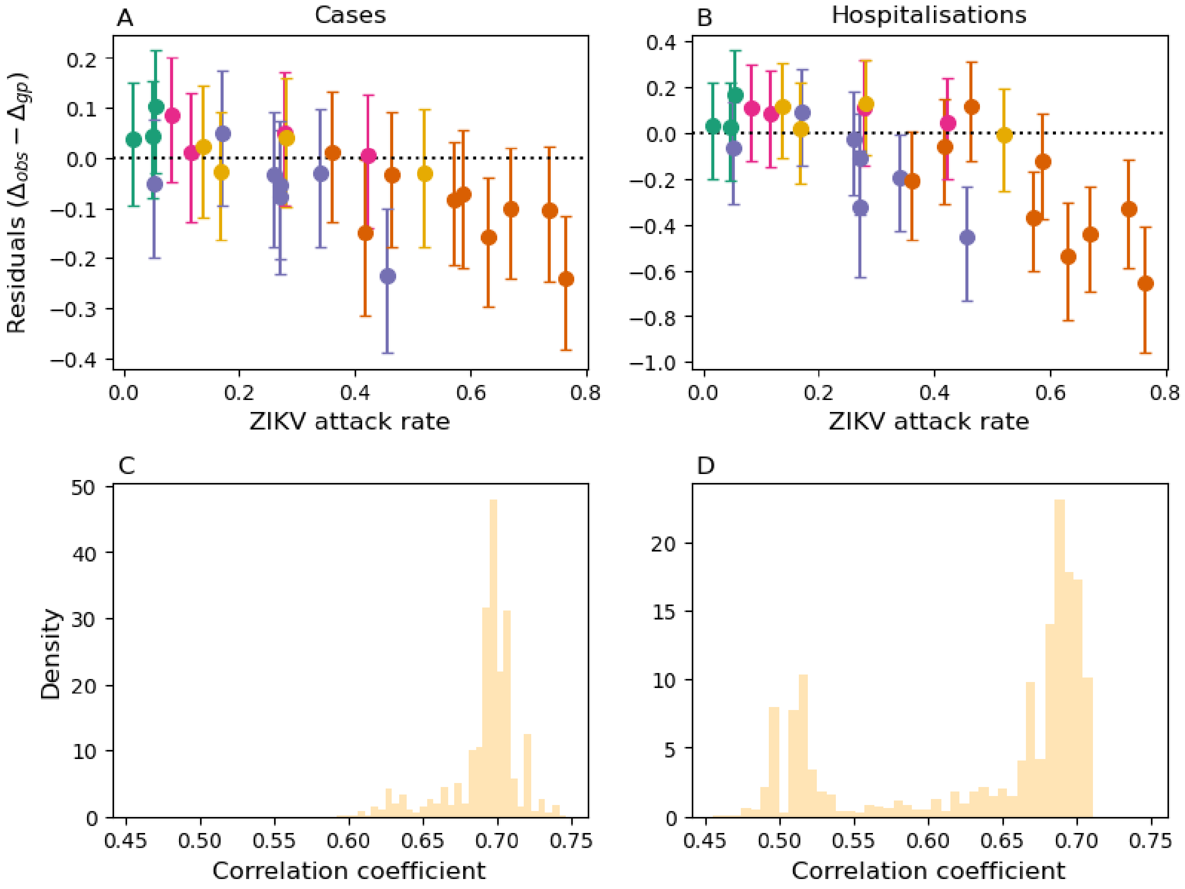

Supplementary Figure 6. **Pre-ZIKV trends of mean age of DENV do not explain the age-shift in 2018-2019.** In this figure we analyse short-term projections from the gaussian process model fitted in Supplementary Fig. 4 and 5 (left and right columns, respectively). More in detail, we use the gaussian process posterior predictive distribution to sample $\bar{A}_{i,2018}$ and $\bar{A}_{i,2019}$ and calculate the age-shift $\Delta_{gp,i}$. This process is repeated 5000 times, and model residuals are calculated by subtracting observed values $\Delta_{obs,i}$. For simplicity, we calculate $\bar{A}_{pre}$ and $\bar{A}_{post}$ by simply taking the mean value of mean age of DENV over the periods 2013-2015 and 2017-2018, respectively. Median values (scatters) and 95% C.I. (error bars) of model residuals for each state are shown in panels A, B. The colour code is the same as in Fig. 3 in the main manuscript. We find that the model overestimates the age-shift in states that experienced large ZIKV outbreaks, and that this discrepancy increases in magnitude with ZIKV attack rate. Panels C, D further elaborate on this trend by showing the distribution of Spearman's rank correlation coefficient between Δ_{gp} and ZIKV attack rates. The model actually predicts a strong positive correlation between these variables, at variance with observations. These results suggest that pre-ZIKV trends of mean age of DENV can not explain age patterns observed during DENV resurgence in 2018 and 2019.

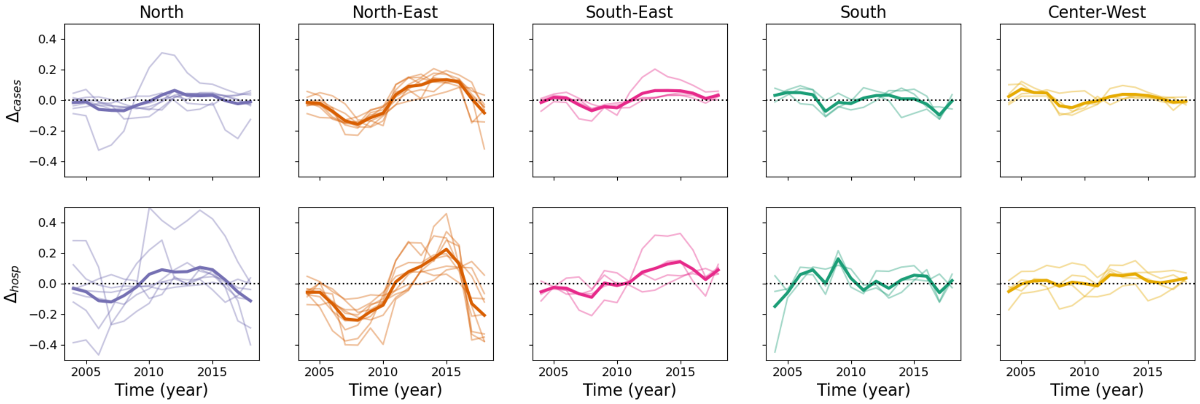

Supplementary Figure 7. **Dynamics of Δ over time.** Panels show Δ for both reported cases (top row) and hospitalizations (bottom row). For each year t we compute $\Delta(t) = (\bar{A}_{post} - \bar{A}_{pre})/\bar{A}_{pre}$, where $\bar{A}_{post}$ aggregates incidence from years t to $t + 1$, and $\bar{A}_{pre}$ aggregates incidence from year $t - 5$ to $t - 3$. For example, $\Delta(2018)$ compares incidence in 2018 and 2019 with incidence from 2013 to 2015. Thick lines represent the average over all states in the same region, while individual states are displayed as dim lines.

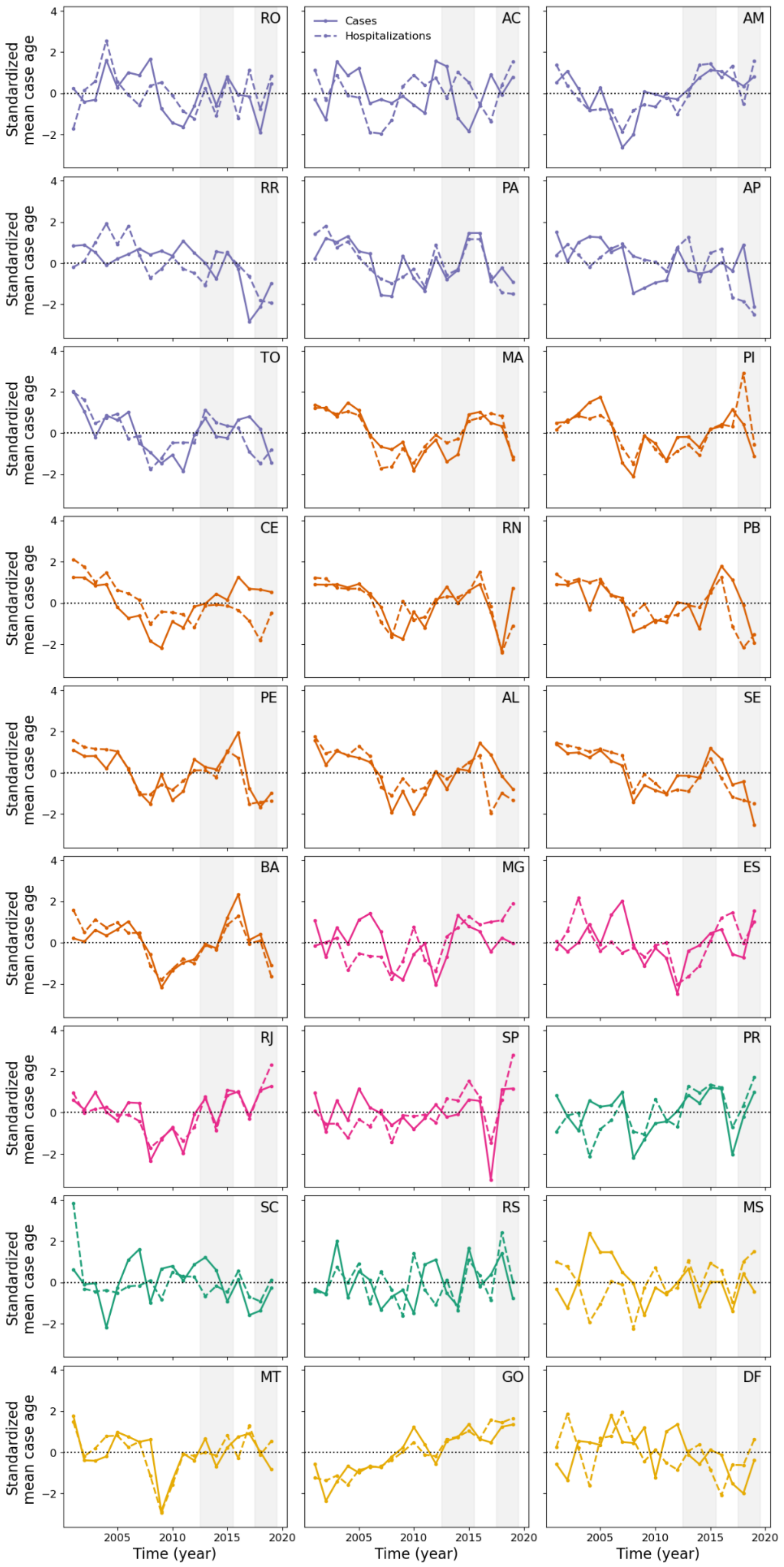

Supplementary Figure 8. **Evolution of $\bar{A}$ over time across Brazil.** Each panel displays $\bar{A}$ for notified DENV cases (solid) and hospitalizations (dashed) from 2001 to 2019 for a single state. $\bar{A}$ was standardised by subtracting its mean value and scaled by its standard deviation computed over the same period. Colour code is the same as in Fig. 3 in the main manuscript. Grey shaded areas correspond to pre- and post-ZIKV periods considered in the main analysis.

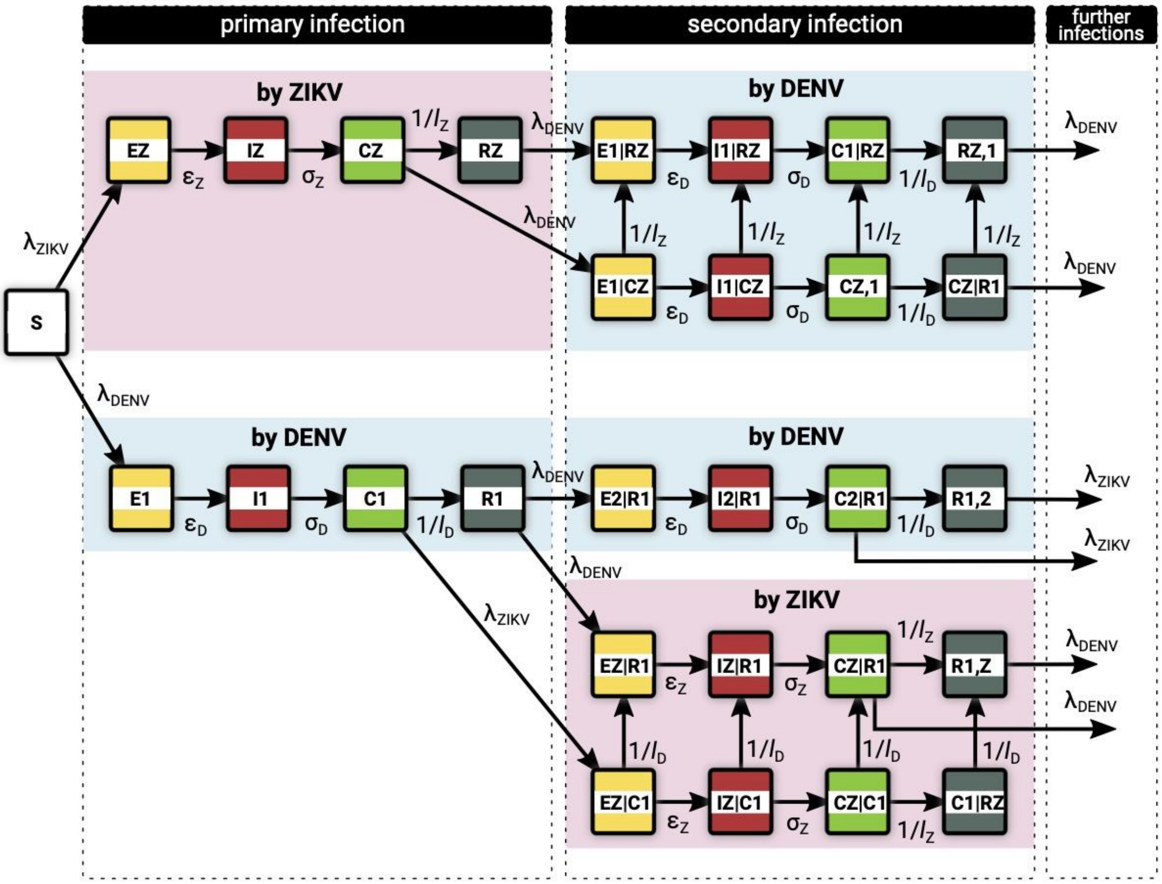

Supplementary Figure 9. **Model diagram.** Each individual is born susceptible (S) and can be infected with ZIKV or any DENV serotype at rates λ_{ZIKV} and λ_{DENV}, respectively (primary infection). The force of infection of each pathogen is proportional to its prevalence and can be affected by seasonality. Following infection with ZIKV (DENV), an individual enters first a latent compartment (E) and then becomes infectious (I) at rate ϵ_Z (ϵ_D). While infectious, recovery occurs at rate σ_Z (σ_D). Recovered individuals first enter a cross-protected compartment (C). Cross-protection is transient and is lost at rate l_Z^{-1}(l_D^{-1}). After that, the host only maintains immunity against reinfection with the same serotype (R). Cross-protection induced by ZIKV (DENV) prevents onward transmission of DENV (ZIKV), but does not affect susceptibility to infection. In addition, DENV-induced cross-protection prevents infections with distinct DENV serotypes. Note that co-infection is not possible in this model.

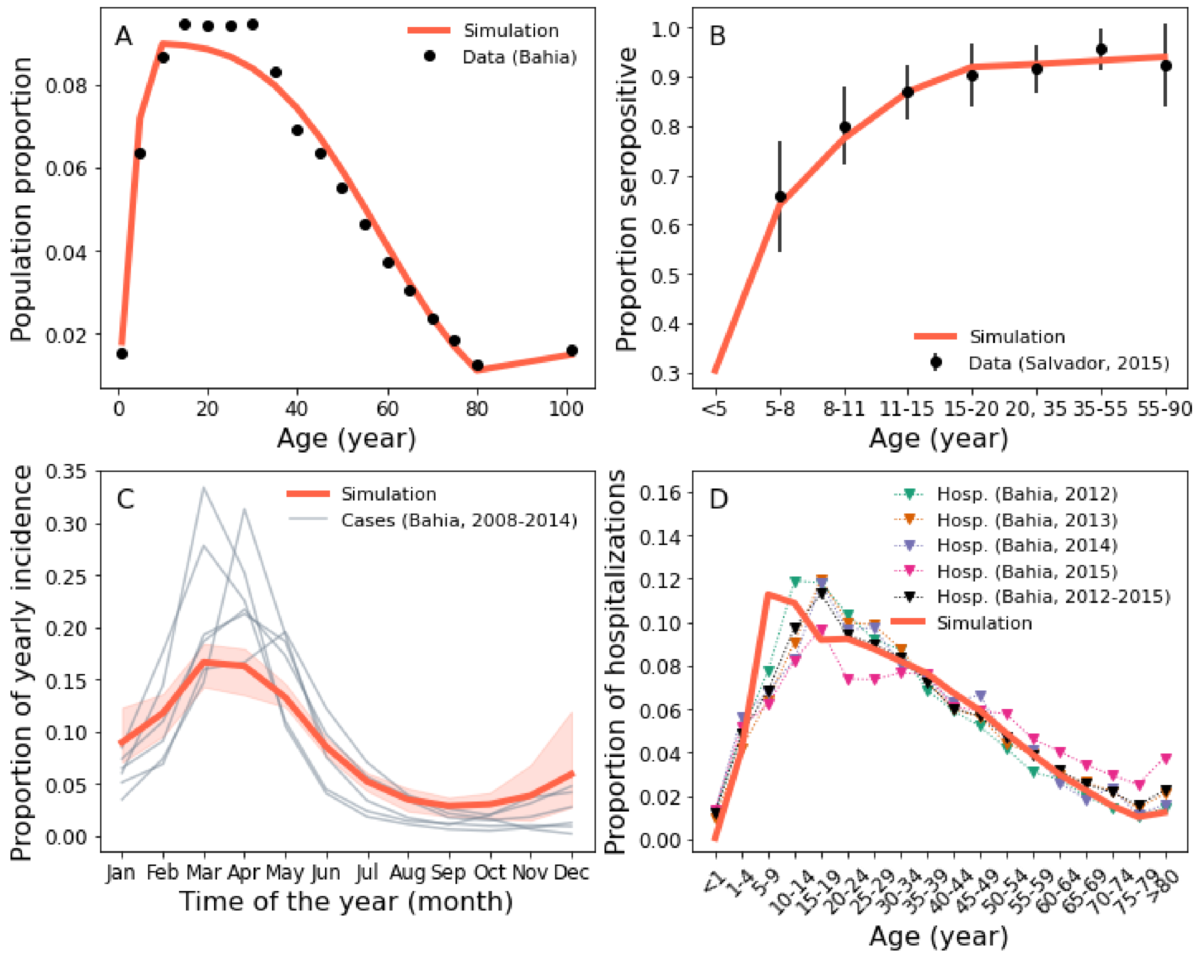

Supplementary Figure 10. **Calibrating host population and DENV endemic dynamics.** (A) Simulated (line) and empirical (dots) population age distributions. The latter was obtained by averaging over Bahia age distributions from 2008 to 2018. (B) Simulated (line) vs observed (dots) seroprevalence profiles. The simulated seroprevalence profile is based on a non-homogeneous Poisson process with intensities $\lambda_1 = 0.04\,y^{-1}$, $\lambda_2 = 0.0014\,y^{-1}$ and a breakpoint $A_0 = 16\,y$. Simulated and empirical profiles are based on findings in [1]. Bars represent the 95% C.I., based on a binomial distribution assumption. (C) Simulated cases (red) vs observed incident Dengue hospitalizations (gray, notified DENV cases) in Bahia from 2008 to 2014. (D) Age distribution of secondary DENV infections in simulations (red line) and observed hospitalizations in Bahia from 2012 to 2015 (markers). In C and D incidence is obtained from the fourth and the first year since the beginning of simulations, respectively.

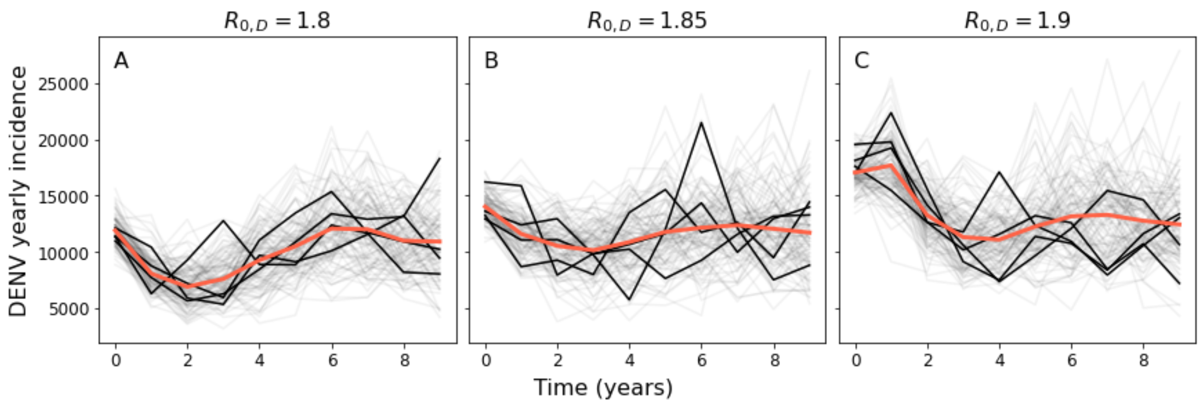

Supplementary Figure 11. **Calibrating $R_{0,D}$.** Panels show DENV yearly incidence from 100 simulations (dim lines) for increasing values of $R_{0,D}$. A few simulations are highlighted in black, while the mean trend is shown in red. Here, the host population and DENV parameters are set as in Supplementary Fig. 10. In particular, individual immune histories are initialised according to the scheme outlined in Supplementary Fig. 10. At endemicity, one would expect yearly incidence to fluctuate around a stationary baseline value, as recovered in the middle panel. Selecting a too small or too large value of $R_{0,D}$ would instead introduce some 'distortion' in DENV dynamics (A,C) because of a mismatch between $R_{0,D}$ and the initial level of immunity implied by the imposed initial serological profile. Please note that it is not possible to recover the exact serological profile in Supplementary Fig. 10B by simply simulating DENV dynamics using a fixed $R_{0,D}$. Nonetheless, this is not a major concern in our analysis since we are only interested in a short time horizon after ZIKV introduction.

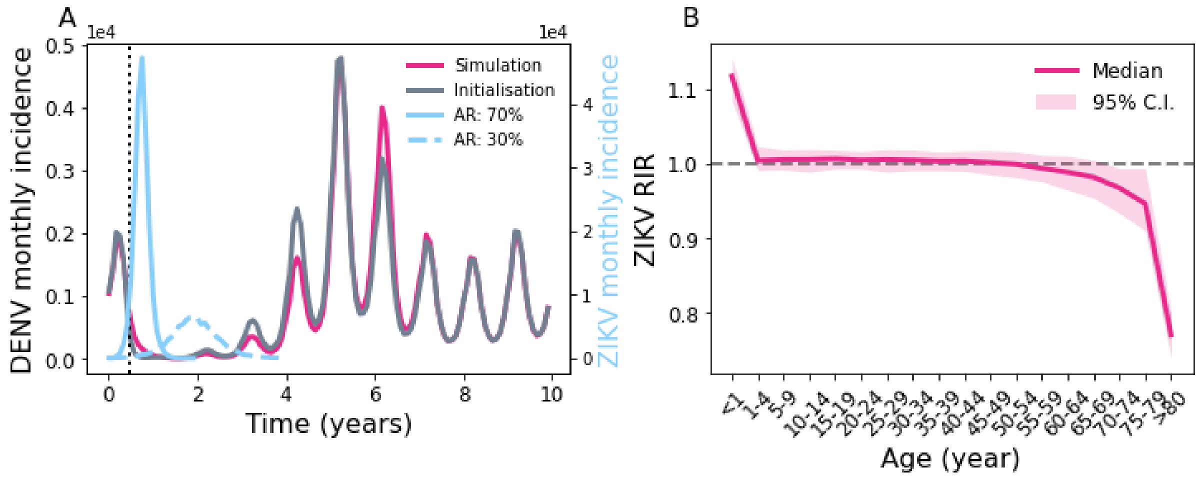

Supplementary Figure 12. **Comparison of ZIKV seeding methods.** (A) Median monthly DENV (grey or fuchsia) incidence over 10 years. With the serology-aware initialisation method (grey line, see also the Supplementary Methods), a proportion $\rho_Z = 0.7$ of the population becomes suddenly infected with ZIKV at $t = 0.45$ years (vertical line), but onward viral transmission is not allowed. This is compared with another simulation method where ZIKV infects roughly the same amount of the population starting from just 10 infectious seeds selected at $t = 0$ (fuchsia). Here, the desired attack rate is obtained by inverting the final epidemic size formula for a SIR model, namely $R_{0,Z} = \; - log(1 - \rho_Z)/\rho_Z \;^2$, and assuming no seasonality in ZIKV transmission ($\beta_{1,Z} = 0$). The same panel also shows ZIKV incidence for two simulations where 70% and 30% of the population becomes infected (solid and dashed blue lines, respectively). Note that the second epidemic is slower and unfolds over multiple years, highlighting technical problems encountered in simulating epidemics with low $R_{0,D}$. (B) ZIKV Relative Infection Risk (median and 95% IQR) using the simulation method to seed ZIKV. The flat profile implies a uniform attack rate over most age classes, which is also implied by the serology-aware initialisation method by construction. In both scenarios, $l_Z = 1$ y. DENV is allowed to spread for a burn-in period of 100 years before the period shown in the plot and $R_{0,D} = 1.65$. Other parameters are as in the default scenario. Results are averaged over 200 simulations.

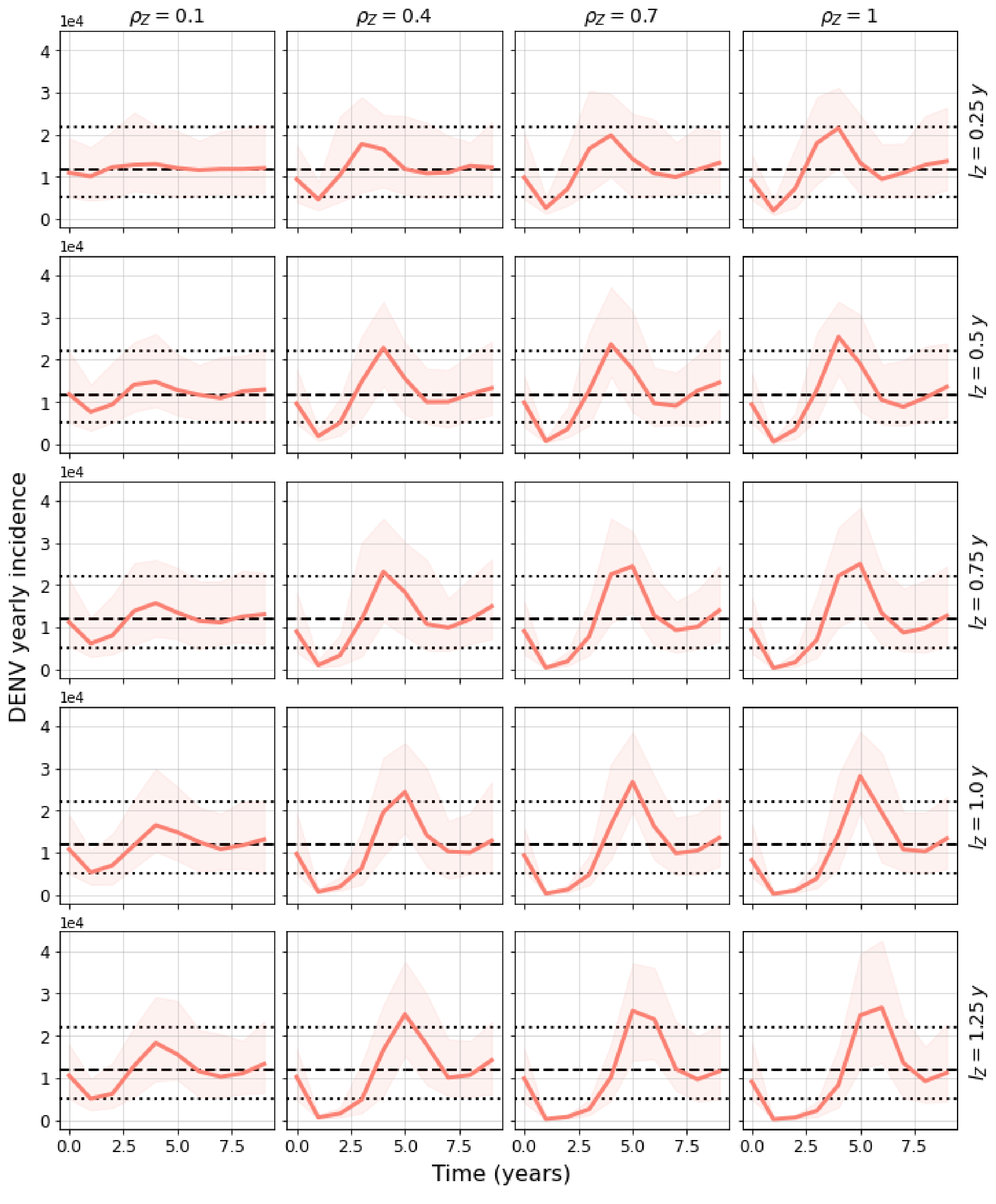

Supplementary Figure 13. **Impact of ZIKV on DENV resurgence.** Each panel shows median DENV yearly incidence (solid red line) over 10 years, ZIKV being introduced at $t = 0.45$ y, for different ZIKV attack rates (increasing from left to right) and duration of ZIKV-induced cross-protection (increasing from top to bottom). The shaded area represents the 95% incidence interquantile range computed from 200 simulations. Simulations show that temporary cross-immunity induced by ZIKV can drive DENV incidence to abnormally low levels with respect to a scenario in which ZIKV never occurred. Median and 95% interquantile range DENV incidence in such baseline scenarios are shown as dashed and dotted lines, respectively. DENV circulates at low levels from 2 to 3 years depending on ZIKV attack rate (ρ_Z) and duration of cross-protection (l_Z), in agreement with a previous simulation study [5]. To obtain these plots, we first allow DENV to spread for a burn-in period of 100 prior to $t = 0$. ZIKV is introduced by infecting a proportion ρ_Z of hosts, as detailed in the main text. Here, $R_{0,D} = 1.65$. Other parameters are set to default values.

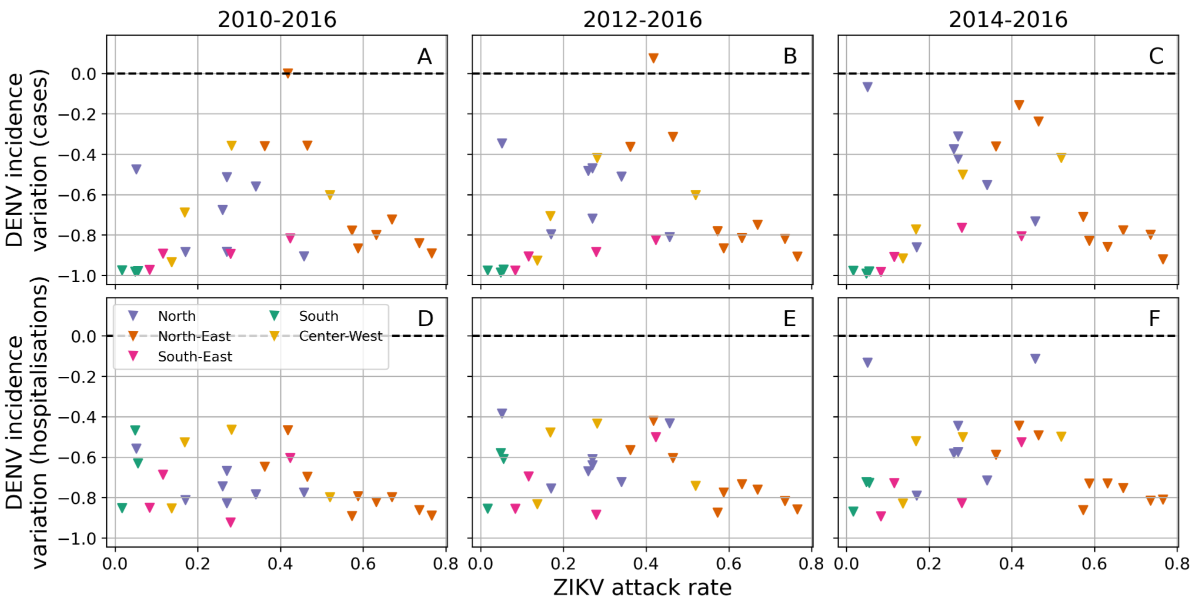

Supplementary Figure 14**. Reduction in DENV incidence following the emergence of ZIKV.** In each panel, scatters represent state-specific relative variation in mean DENV yearly incidence before and after ZIKV against estimated ZIKV attack rate. First and second rows are based on reported cases and hospitalisations, respectively. More in detail, we compare DENV incidence in 2017 with incidence from three different periods, namely 2010-2016 (A,D), 2012-2016 (B,E) and 2014-2016 (C,F). Almost all states experienced a reduction in both DENV reported cases and hospitalisations in 2017 with respect to previous years, although no firm conclusion on the relationship between DENV reduction and ZIKV attack rate can be drawn based on these data. Interestingly, DENV transmission was particularly disrupted in states with either low or high ZIKV attack rates. It should be noted, however, that historical trends of DENV circulation may vary considerably from state to state [6], potentially confounding our analysis.

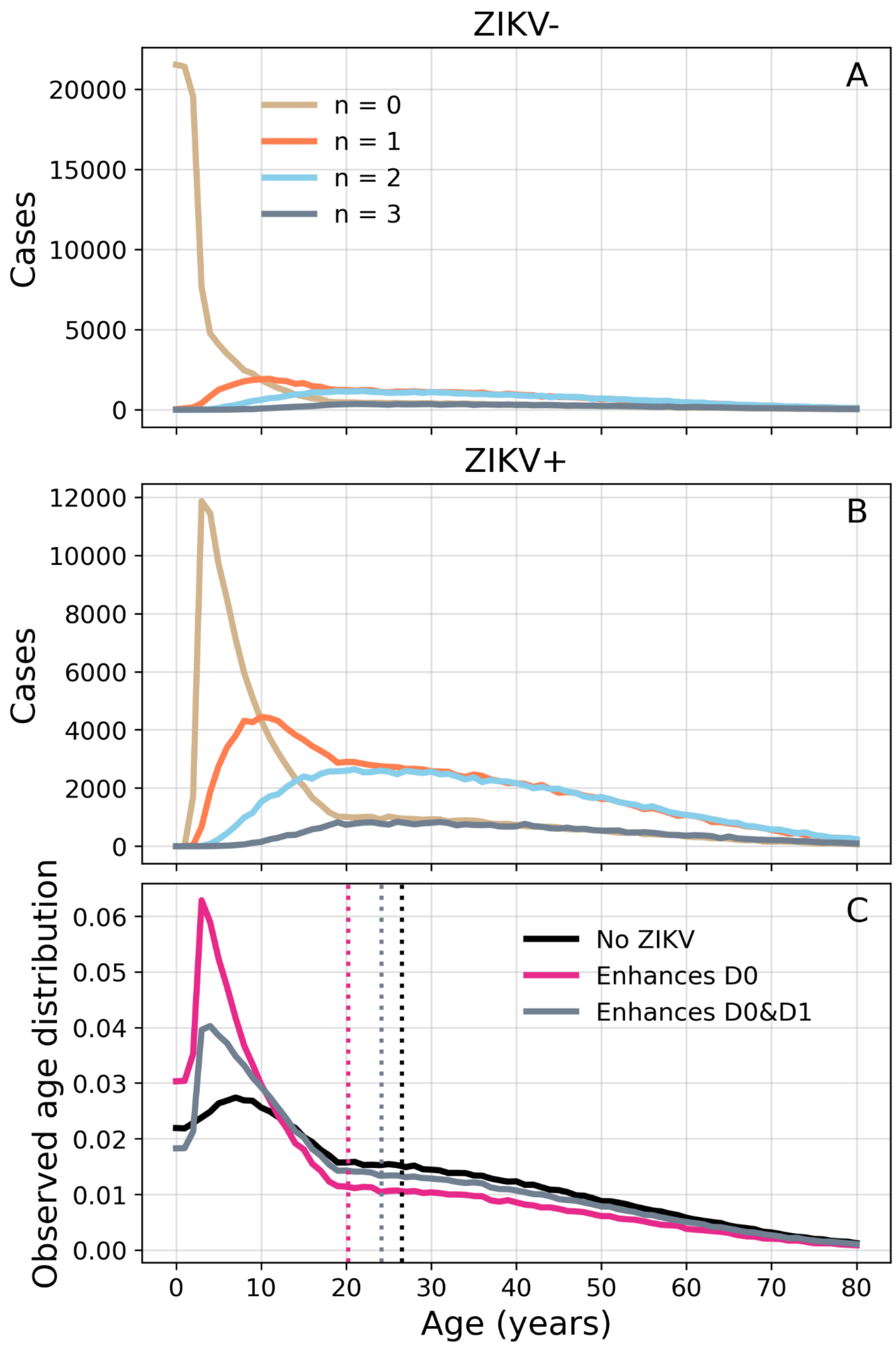

Supplementary Figure 15. **DENV seroincidence.** Counts of incident DENV cases by number $n = 0, 1, 2, 3$ of past DENV infections in ZIKV- (A) and ZIKV+ (B) hosts, assuming a 70% ZIKV attack rate. Case counts are collected during the fourth year since simulation start time and aggregated over multiple simulations. (C) Distribution of observed incident DENV cases, assuming that ZIKV enhances severity in primary DENV infections only (fuchsia, mechanism (1) in the main manuscript) and both primary and secondary infections (dark grey, mechanism (2) in the main manuscript). Dotted lines represent the mean observed case age from the corresponding distribution. (C) demonstrates that mechanism (1) is able to generate a larger decrease in observed case age than mechanism (2), with respect to a reference scenario with no ZIKV (black). Host population and transmission parameters are set to default values. The underlying explanation is that secondary DENV infections in ZIKV+ hosts (orange lines in (A,B)), whose severity is enhanced by mechanism (2) but not (1), typically affect older hosts than primary DENV infections (beige lines). Overall, both mechanisms can lower the mean observed case age.

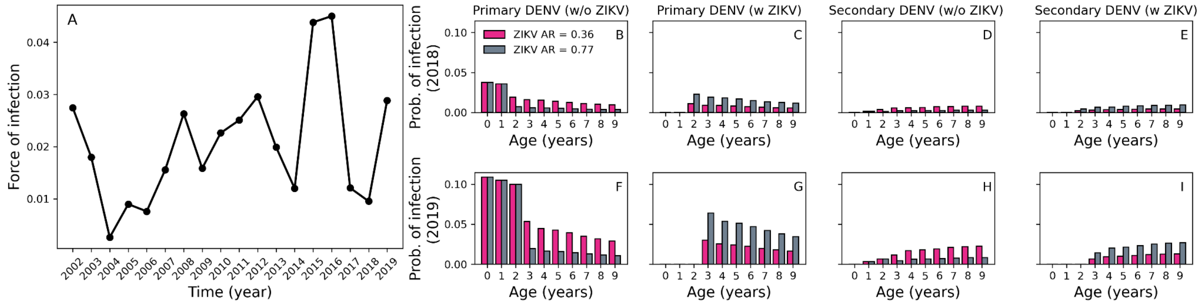

Supplementary Figure 16. **Reconstructing seroincidence in children in the Northeast.** Yearly estimates of the force of infection λ_t of DENV from $t = 2002$ to 2019 in the Northeast of Brazil. We recovered λ_t from the second panel in Fig. 3 in Brito et al.[6] using *PlotDigitizer*[7]. (B-I) Probability of primary and secondary DENV infections in children up to 9 years old in 2018 (B-E) or 2019 (F-I). Fuchsia and gray bars correspond, respectively, to scenarios where the ZIKV attack rate is 0.36 and 0.77, i.e. the extremal values reported among Northeastern states (see Fig. 3 in the main manuscript). Panels (B-I) show that primary DENV infections are more common than secondary infections in ZIKV-positive children in areas with high ZIKV transmission. We calculated infection probabilities by adapting the methodology presented in Brito et al.[6]. Let $P_{a,t}(D|Z = 0, 1)$ denote the probability of primary ($D = 1$) or secondary ($D = 2$) DENV infections in hosts aged a years old in year t, conditional on $Z = 0, 1$ past ZIKV infections. For $D = 1$: $P_{a,t}(D = 1|Z) = \left(1 - e^{-4\lambda_t}\right) \cdot d^{(0)}_{a,t} \cdot z^{Z}_{a,t} \cdot \left(1 - z_{a,t}\right)^{1-Z}$, where $d^{(m)}_{a,t}$ is the probability of having experienced exactly m DENV infections at age a and $z_{a,t}$ is the probability of having experienced a ZIKV infection by age a in year t. We assume that $z_{a,t}$ equals ZIKV attack rate for hosts born before 2016 (included), and is 0 otherwise. This assumption implies uniform attack rates across age (as assumed in the main manuscript). $P_{a,t}(D = 2|Z)$ is defined similarly, with transmission intensity $1 - e^{-3\lambda_t}$ and $d^{(0)}_{a,t}$ being replaced by $d^{(1)}_{a,t}$. We calculate $d^{(m=0,1)}_{a,t}$ as $d^{(0)}_{a,t} = \prod_{i=t-a}^{t-1} e^{-4\lambda_i}$ and $d^{(1)}_{a,t} = \sum_{j=t-a}^{t-1} \left(\prod_{i=t-a}^{j-1} e^{-4\lambda_i}\right)\left(1 - e^{-3\lambda_j}\right)\left(\prod_{i=j+1}^{t-1} e^{-3\lambda_i}\right)$ for $a > 0$ and $d^{(0)}_{0,t} = 1$, $d^{(1)}_{0,t} = 0$.

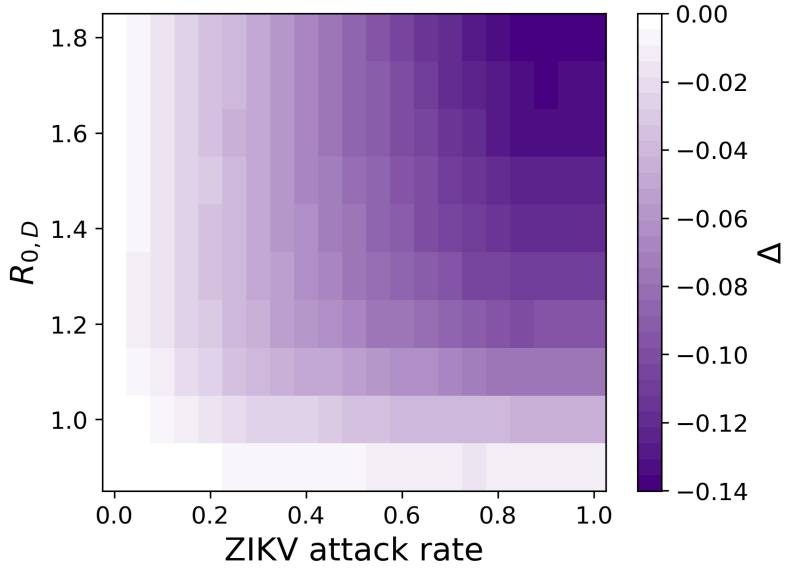

Supplementary Figure 17**. Mean age-shift under different ZIKV and DENV transmission scenarios.** The heatmap mirrors Fig. 4C in the main text under the assumption that ZIKV enhances both primary and secondary DENV infections (mechanism (2) in the main text). Incidence weights $w_n^{-/+}$ are the same as those specified in Fig. 4 in the main manuscript for mechanism (2). Δ is calculated using incidence from the fourth year since simulation start time. DENV is allowed to spread for a burn-in period of 100 years before ZIKV is introduced. We average results over 300 independent realisations.

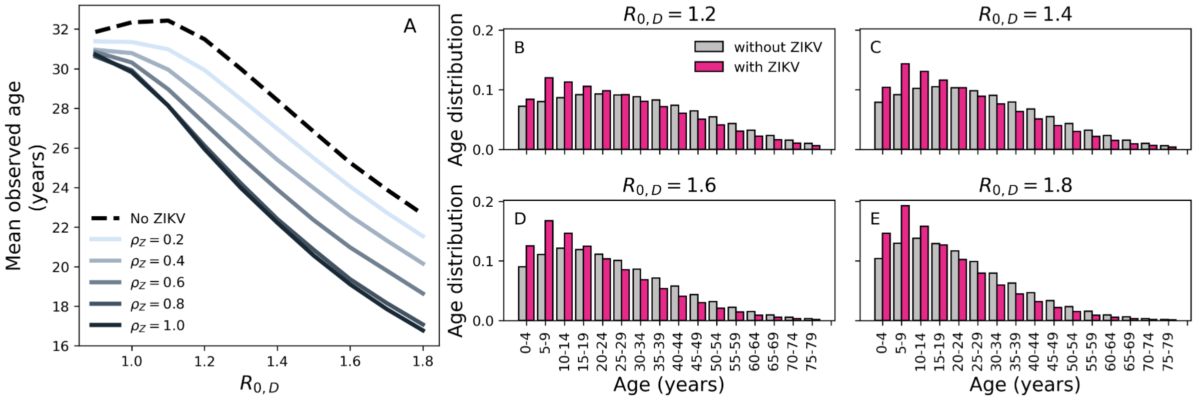

Supplementary Figure 18. **Impact of baseline DENV transmission.** (A) Mean observed age of incident cases as a function of $R_{0,D}$ and for different values of ZIKV attack rate ($\bar{A}_{post}$). The dashed line corresponds to simulations without ZIKV and is thus equivalent to $\bar{A}_{pre}$, the mean observed case age before ZIKV is introduced. The mean observed case age before ($\bar{A}_{pre}$) and after ($\bar{A}_{post}$) ZIKV, which enter Δ's definition through $\Delta = \bar{A}_{post}/\bar{A}_{pre} - 1$, do not depend on $R_{0,D}$ in the same way. Typically, $\bar{A}_{post}$ decreases with $R_{0,D}$ (solid lines), while $\bar{A}_{pre}$ initially increases with $R_{0,D}$ and then declines (dashed line). Taken together, these results mean that Δ must decrease more rapidly at low $R_{0,D}$. (B-E) Age distributions of reported DENV cases for different values of $R_{0,D}$, without ZIKV (grey) and with a ZIKV epidemic infecting a proportion $\rho_Z = 0.6$ of the host population (fuchsia). Panels (B-E) show that the effect of ZIKV-induced modulation of disease would be to shift the entire distribution to younger ages; moreover, the age group from 5 to 15 years is always the modal one. Results are based on the assumption that ZIKV enhances disease in primary DENV infections only (mechanism (1) in the main text). To obtain these plots, we let DENV spread for a burn-in period of 100 years before ZIKV is introduced at $t = 0.45$ y. We compute DENV incidence during the fourth year since ZIKV emergence and average results over 100 independent simulations. Other parameters are set to default values.

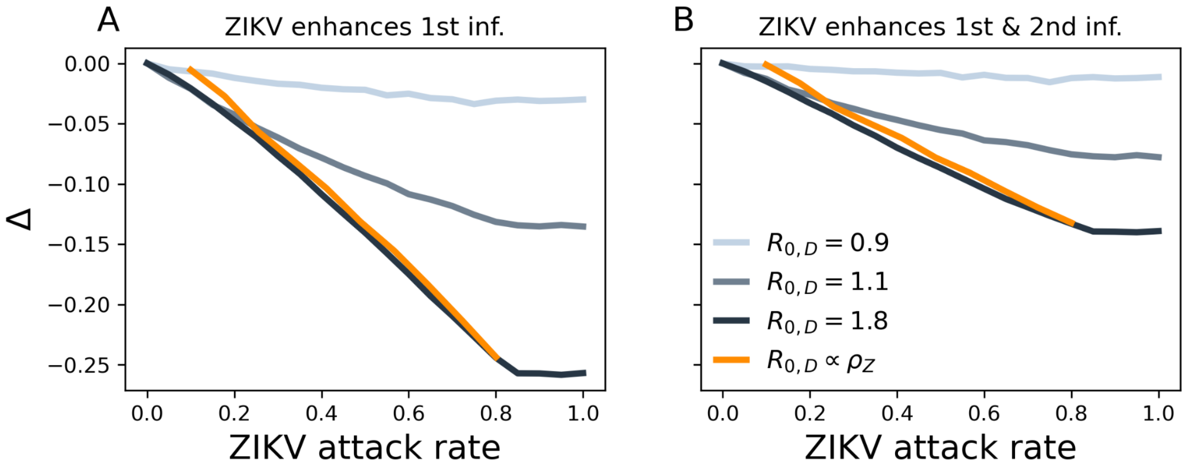

Supplementary Figure 19. **Average age-shift under different DENV transmission scenarios.** Panels show Δ as a function of ZIKV attack rate, under the assumptions that ZIKV enhances primary DENV infections only (A) or both primary and secondary infections (B). Incidence weights $w_n^{-/+}$ are the same as those specified in Fig. 4 in the main manuscript. Grey lines correspond to scenarios where $R_{0,D}$ is held constant as ρ_Z is varied. In contrast, the orange line corresponds to a scenario where $R_{0,D}$ and ρ_Z co-vary due to shared drivers of transmission. In particular, we assume a linear relationship: $R_{0,D} = 1.29(\rho_Z - 0.1) + 0.9$, where ρ_Z ranges from 0.1 to 0.8. In this scenario, values of Δ are comparable to those observed under high DENV circulation ($R_{0,D} = 1.8$). Of note, the relation between Δ and ρ_Z is steeper in this case, meaning that co-variation in transmission can exacerbate the effect of ZIKV on Δ. In addition, the steeper slope implies that Δ hits 0 faster as ρ_Z is decreased. Here, we let DENV spread for 100 years before introducing ZIKV and calculate Δ using incidence collected during the fourth year since ZIKV emergence. Other parameters are the same as in Supplementary Fig. 10. Results are averaged over 300 simulations.

Supplementary references

1. Rodriguez-Barraquer, I. *et al.* Impact of preexisting dengue immunity on Zika virus emergence in a dengue endemic region. *Science* **363**, 607–610 (2019).
2. Miller, J. C. A note on the derivation of epidemic final sizes. *Bull. Math. Biol.* **74**, 2125–2141 (2012).
3. Rasmussen, C. E. & Williams, C. K. I. *Gaussian Processes for Machine Learning*. (The MIT Press, 2005). doi:10.7551/mitpress/3206.001.0001.
4. Jordahl, K. GeoPandas: Python tools for geographic data. (2023).
5. Borchering, R. K. *et al.* Impacts of Zika emergence in Latin America on endemic dengue transmission. *Nat. Commun.* **10**, 5730 (2019).
6. Brito, A. F. *et al.* Lying in wait: the resurgence of dengue virus after the Zika epidemic in Brazil. *Nat. Commun.* **12**, 2619 (2021).
7. PlotDigitizer: Extract Data from Graph Image Online. *PlotDigitizer* https://plotdigitizer.com/.
