# Supplementary material for: Shifting patterns of dengue three years after Zika virus emergence in Brazil
Source: Nat Commun. 2024 Jan 20;15:632. doi: 10.1038/s41467-024-44799-x (PMC10799945; doi:10.1038/s41467-024-44799-x)
Supplement: Supplementary file 2 — Reporting Summary [file 41467_2024_44799_MOESM2_ESM.pdf]

nature portfolio

Corresponding author(s): Francesco Pinotti

Last updated by author(s): Dec 15, 2023

# Reporting Summary

Nature Portfolio wishes to improve the reproducibility of the work that we publish. This form provides structure for consistency and transparency in reporting. For further information on Nature Portfolio policies, see our Editorial Policies and the Editorial Policy Checklist.

## Statistics

For all statistical analyses, confirm that the following items are present in the figure legend, table legend, main text, or Methods section.

| n/a | Confirmed | |
|---|---|---|
| ☐ | ☒ | The exact sample size (*n*) for each experimental group/condition, given as a discrete number and unit of measurement |
| ☒ | ☐ | A statement on whether measurements were taken from distinct samples or whether the same sample was measured repeatedly |
| ☐ | ☒ | The statistical test(s) used AND whether they are one- or two-sided<br>*Only common tests should be described solely by name; describe more complex techniques in the Methods section.* |
| ☐ | ☒ | A description of all covariates tested |
| ☒ | ☐ | A description of any assumptions or corrections, such as tests of normality and adjustment for multiple comparisons |
| ☐ | ☒ | A full description of the statistical parameters including central tendency (e.g. means) or other basic estimates (e.g. regression coefficient) AND variation (e.g. standard deviation) or associated estimates of uncertainty (e.g. confidence intervals) |
| ☐ | ☒ | For null hypothesis testing, the test statistic (e.g. *F*, *t*, *r*) with confidence intervals, effect sizes, degrees of freedom and *P* value noted<br>*Give P values as exact values whenever suitable.* |
| ☐ | ☒ | For Bayesian analysis, information on the choice of priors and Markov chain Monte Carlo settings |
| ☒ | ☐ | For hierarchical and complex designs, identification of the appropriate level for tests and full reporting of outcomes |
| ☐ | ☒ | Estimates of effect sizes (e.g. Cohen's *d*, Pearson's *r*), indicating how they were calculated |

*Our web collection on statistics for biologists contains articles on many of the points above.*

## Software and code

Policy information about availability of computer code

| | |
|---|---|
| Data collection | We used Python (v3.8) to clean and merge datasets downloaded from publicly available repositories. |
| Data analysis | We used R (v 4.2.2), Rstudio ("Prairie Trillium" Release (8aaa5d47, 2022-03-17) for macOS) and Python (v3.8) to analyze the data. Maps were realized in Python using the module GeoPandas v0.11.1. Vector Suitability (Index P) was calculated in R using MVSE package v1.01. Linear regression analyses were performed in R using the packages rstanarm v2.21.3 and loo v2.5.1. |

For manuscripts utilizing custom algorithms or software that are central to the research but not yet described in published literature, software must be made available to editors and reviewers. We strongly encourage code deposition in a community repository (e.g. GitHub). See the Nature Portfolio guidelines for submitting code & software for further information.

## Data

Policy information about availability of data

All manuscripts must include a data availability statement. This statement should provide the following information, where applicable:

- Accession codes, unique identifiers, or web links for publicly available datasets
- A description of any restrictions on data availability
- For clinical datasets or third party data, please ensure that the statement adheres to our policy

Counts of probable Dengue virus cases and hospitalizations for individual states and the city of Salvador, Bahia, were obtained from the Brazilian Information System for Notifiable Diseases (SINAN, https://datasus.saude.gov.br/acesso-a-informacao/doencas-e-agravos-de-notificacao-2001-a-2006-sinan and https://

datasus.saude.gov.br/acesso-a-informacao/doencas-e-agravos-de-notificacao-de-2007-em-diante-sinan) and Unified Health System (SUS) through the DATASUS (https://datasus.saude.gov.br/acesso-a-informacao/morbidade-hospitalar-do-sus-sih-sus). Population size estimates were obtained from DATASUS (https://datasus.saude.gov.br/populacao-residente). Weekly DENV and ZIKV notified cases (clinically suspected and confirmed) in the Bahian city of Feira de Santana were obtained directly from the Secretaria Municipal de Saúde of the city (https://www.feiradesantana.ba.gov.br/servicos.asp?id=14&link=sms/vigilancia_saude/vigilancia_epidemiologica.asp). All relevant data used in Fig. 1-3 and Supplementary Fig. 1-8,14,16 are available in the GitHub repository https://github.com/francescopinotti92/dengue_and_zika_brazil.

## Research involving human participants, their data, or biological material

Policy information about studies with human participants or human data. See also policy information about sex, gender (identity/presentation), and sexual orientation and race, ethnicity and racism.

| | |
|---|---|
| Reporting on sex and gender | Sex and gender variables were not included in this study. |
| Reporting on race, ethnicity, or other socially relevant groupings | No variables of this kind were included in this study. |
| Population characteristics | The present study used publicly available population-level data for Brazil disaggregated by state, age group and year. |
| Recruitment | This was an observational study that did not involve recruitment of participants. |
| Ethics oversight | The ethics approval was not required as the present study used publicly available population-level data. |

Note that full information on the approval of the study protocol must also be provided in the manuscript.

# Field-specific reporting

Please select the one below that is the best fit for your research. If you are not sure, read the appropriate sections before making your selection.

☐ Life sciences ☐ Behavioural & social sciences ☒ Ecological, evolutionary & environmental sciences

For a reference copy of the document with all sections, see nature.com/documents/nr-reporting-summary-flat.pdf

# Ecological, evolutionary & environmental sciences study design

All studies must disclose on these points even when the disclosure is negative.

| | |
|---|---|
| Study description | In this study we used publicly available case and hospitalization counts associated with dengue virus to investigate its changing epidemiology in the post-Zika virus era in Brazil. Linear and Gaussian-process regression methods were used to assess relationships between the observed shift in the age of reported dengue cases before and after Zika virus emergence and ecological and epidemiological factors. There was no assignment to treatment. We used numerical simulations to test whether cross-reactive interactions between Zika and Dengue virus could explain observed patterns. |
| Research sample | The present study used publicly available population-level data for Brazil disaggregated by state, age group and year. |
| Sampling strategy | Not applicable. The present study used publicly available population-level data for Brazil. |
| Data collection | None of the authors were present during sample collection. The present study used publicly available population-level data for Brazil. Case and hospitalization counts were collected by Ministerio da Saude Brasil through passive health surveillance. F.P. and M.M.L. downloaded and collated case and hospitalisation counts from the Ministerio da Saude Brasil. Data sources are listed in "Data Availability". |
| Timing and spatial scale | This study includes dengue virus cases and hospitalizations from 2001 to 2019 in Brazil. Information about circulating serotypes was only available starting from 2014. The Ministry of Health updates its public repository on a yearly basis. Data was downloaded from the relevant repositories in November 2022. |
| Data exclusions | No data was excluded from this study. |
| Reproducibility | Our study involved only numerical experiments. Relevant code has been provided to ensure reproducibility of numerical simulations and data analysis. |
| Randomization | This was an observational study that made use of publicly available data and did not make any assignments to groups. |
| Blinding | No blinding was necessary since this study involved only counts of reported DENV and ZIKV cases aggregated by age. |

Did the study involve field work? ☐ Yes ☒ No

# Reporting for specific materials, systems and methods

We require information from authors about some types of materials, experimental systems and methods used in many studies. Here, indicate whether each material, system or method listed is relevant to your study. If you are not sure if a list item applies to your research, read the appropriate section before selecting a response.

## Materials & experimental systems

| n/a | Involved in the study |
|---|---|
| ☒ | ☐ Antibodies |
| ☒ | ☐ Eukaryotic cell lines |
| ☒ | ☐ Palaeontology and archaeology |
| ☒ | ☐ Animals and other organisms |
| ☒ | ☐ Clinical data |
| ☒ | ☐ Dual use research of concern |
| ☒ | ☐ Plants |

## Methods

| n/a | Involved in the study |
|---|---|
| ☒ | ☐ ChIP-seq |
| ☒ | ☐ Flow cytometry |
| ☒ | ☐ MRI-based neuroimaging |
